# Bimodal onset and pan-cancer uniformity of immune-mediated liver injury: a retrospective cohort study

**DOI:** 10.3389/fimmu.2025.1612287

**Published:** 2025-07-02

**Authors:** Jiaojiao Song, Biying Xu, Lan Yu, Haiwei Fu, Binliang Wang, Mo Zhou, Yumin Hu, Yang Xia

**Affiliations:** ^1^ Department of Respiratory and Critical Care Medicine, The First People’s Hospital of Linhai, Taizhou, Zhejiang, China; ^2^ Department of Respiratory and Critical Care Medicine, Second Affiliated Hospital, Zhejiang University School of Medicine, Hangzhou, Zhejiang, China; ^3^ Department of Respiratory and Critical Care Medicine, Taizhou First People’s Hospital, Taizhou, Zhejiang, China

**Keywords:** immune-mediated liver injury, PD-1/PD-L1 inhibitor, clinical characteristic, immune dysregulation, management

## Abstract

**Background:**

Immune-mediated liver injury (IMLI) is a critical adverse event in patients treated with PD-1/PD-L1 inhibitors. The study aims to characterize the clinical heterogeneity, temporal dynamics, and immunological drivers of PD-1/PD-L1 inhibitor-associated IMLI and optimize surveillance and management strategies.

**Methods:**

We retrospectively recruited 373 IMLI patients. We evaluated clinical data, including liver injury patterns, severity, temporal trends, and immune cell subsets. Statistical analyses identified risk factors for severe IMLI and temporal dynamics.

**Results:**

Among 373 patients (median age: 65 years; male: 74.8%), IMLI severity was graded as G1 (53.9%), G2 (25.2%), G3 (17.9%), and G4 (2.7%), with hepatocellular (17.2%), mixed (42.6%), and cholestatic (40.2%) patterns observed. The median time to onset was 106–115 days across severity groups. In contrast, recovery time was significantly prolonged (G1/2: 14 days vs. G3/4: 23 days, *P*<0.05), and recovery-phase CD8^+^ T cells (524.9 vs. 270.68 cells/μL, *P*=0.026) were higher in severe cases. Bimodal onset peaks occurred at 1–2 months and 3–4 months, with 88% recovering within 100 days. No tumor-type differences existed in patterns (*P*=0.427) or severity (*P*=0.054). Elevated baseline NK cells (*OR*=1.004, *P*=0.036) predicted severe IMLI.

**Conclusions:**

IMLI demonstrates bimodal onset and pan-cancer uniformity, driven by systemic immune dysregulation. Baseline NK cells are potential predictors of severity. Risk-adapted monitoring within 4 months post-ICI and standardized protocols are recommended.

## 1 Introduction

The advent of programmed cell death protein 1/programmed death-ligand 1 (PD-1/PD-L1) inhibitors has revolutionized cancer treatment by reinvigorating antitumor immunity (1, 2), yet their clinical utility is constrained by immune-related adverse events (irAEs) (3), particularly immune-mediated liver injury (IMLI), due to its heterogeneous presentation and potentially life-threatening consequences. Cumulative evidence indicates that IMLI occurs in 2-25% of patients receiving immune checkpoint inhibitors (ICIs), with mortality rates reaching 22% in severe cases (4, 5), which often necessitates treatment interruption and compromises antitumor efficacy and deteriorates patient-reported quality of life (6). The risk of IMLI exhibits significant variation depending on ICI types (7). Clinical trial data demonstrate a substantially higher incidence of CTLA-4 inhibitor-associated IMLI (2%-15%) compared to PD-1 inhibitors (0%-3%) and PD-L1 inhibitors (0%-6%) (8, 9). Importantly, real-world evidence reveals an amplified risk profile, particularly in combination therapy cohorts, which shows both increased frequency and severity of hepatic adverse events (10).

While current guidelines (ESMO/ASCO) provide a framework for managing irAEs based on Common Terminology Criteria for Adverse Events (CTCAE) grading (4, 11), critical knowledge gaps impede risk-benefit optimization. First, the diagnosis of IMLI relies on nonspecific biochemical markers and the exclusion of alternative etiologies, lacking validated biomarkers for early detection (12). Second, the temporal heterogeneity of IMLI onset remains poorly characterized, with prior studies reporting a unimodal distribution (median 6–14 weeks, but with significant variation-latencies of as much as 93 weeks have been reported (13), failing to capture nuanced temporal dynamics such as bimodal peaks or recovery trajectories. Third, emerging evidence indicates that hepatocellular carcinoma (HCC) patients exhibit a modestly higher incidence of IMLI following ICI therapy compared to other cancer types (14, 15). This observed disparity may be associated with underlying chronic liver disease or prior hepatic interventions. Besides, clinical observations have identified similarly elevated IMLI incidence rates and greater disease severity in melanoma and renal cell carcinoma (RCC) cohorts (10). Debates persist regarding whether IMLI features are tumor-agnostic or modulated by cancer-specific microenvironments, with implications for universal versus tailored management strategies (16–18).

To address these gaps, we aimed to delineate the temporal dynamics and immunological drivers of IMLI, evaluate its pan-cancer uniformity, and identify biomarkers for risk stratification. By integrating clinical profiling with peripheral immune monitoring, this study provides a roadmap for optimizing surveillance and therapeutic protocols.

## 2 Materials and methods

### 2.1 Study population

This retrospective cohort study enrolled consecutive patients admitted to the Second Affiliated Hospital of Zhejiang University School of Medicine between January, 2020 and July, 2024. Inclusion criteria were as follows: (i) adult patients (≥18 years) with pathologically confirmed malignancies; (ii) receipt of ICI therapy; (iii) Patients with normal baseline liver function who developed abnormalities during treatment, or patients with baseline liver function exceeding the normal range who exhibited significant biochemical abnormalities during treatment; (iv) Patients receiving antiviral therapy with hepatitis B virus DNA titers <100 IU/mL (if co-infected with hepatitis B) (19). Exclusion criteria: (i) Liver function abnormalities attributed to other causes (e.g., active viral hepatitis, tumor liver metastasis progression, hepatic hypoperfusion); (ii) Lack of clinical data.

### 2.2 Data collection

Demographic data (age, sex, BMI, comorbidities), tumor type, and treatment regimens (ICI type, dosage, duration) were systematically collected. IMLI profiles were also recorded, including time of onset, clinical manifestations, laboratory parameters, imaging/pathological findings, concomitant irAEs, treatment measures, and outcomes. Key laboratory parameters including absolute leukocyte/neutrophil/lymphocyte counts, alanine aminotransferase (ALT), aspartate aminotransferase (AST), alkaline phosphatase (ALP), gamma-glutamyl transferase (GGT), total bilirubin (TBIL), direct bilirubin (DBIL), albumin, coagulation profile, virological serology (hepatitis viruses, CMV, HSV, EBV antibodies), autoimmune markers (antinuclear antibodies, immunoglobulins), and metabolic indicators (ceruloplasmin, ferritin). Two blinded investigators independently cross-validated all data and confirmed IMLI diagnosis through the systematic exclusion of alternative etiologies. Discrepancies were resolved through multidisciplinary consensus involving hepatologists. The R-value [(ALT/ULN)/(ALP/ULN)] was calculated based on the ratio of ALT to the upper limit of normal (ULN) and AST to ULN, categorizing liver injury patterns into hepatocellular (R≥5), mixed (2<R<5), and cholestatic (R ≤ 2). Disease severity was graded according to CTCAE V5.0 (20), with grades 1–2 defined as mild liver injury (G1/2), grades 3–4 as severe liver injury (G3/4), and grade 5 as fatal liver injury. Patients were followed until July, 2024, death, or liver function recovery (defined as ALT/AST ≤1×ULN and ALP ≤1.5×ULN, or return to baseline levels). Follow-up data were censored at the last documented contact for patients lost to follow-up (n=7).

### 2.3 Peripheral blood immune cell subset analysis

Flow cytometry (BD FACSCanto™ II; antibody panel: CD3 FITC, CD4 PE-Cy7, CD8 APC-Cy7, CD16 PE + CD56 PE, CD19 APC, CD45 PerCP-Cy5.5) was performed to analyze peripheral blood immune cell subset counts at three time points (baseline, early phase, and recovery phase). As a routine clinical assay in our hospital, the standardized protocol was strictly followed according to the manufacturer’s instructions. Freshly collected samples were processed and analyzed within 24 hours to minimize the technical and operator-dependent variability. Evaluated populations included total T lymphocytes (CD3^+^), CD4^+^/CD8^+^ T-cell subsets, natural killer (NK) cells (CD3^-^CD16^+^CD56^+^), and B lymphocytes (CD19^+^). Baseline data were defined as laboratory results from the first hospitalization before initiating immunotherapy. The early phase was defined as the period after starting immunotherapy but before the onset of IMLI. The recovery phase was defined as the period when liver function returned to normal or baseline levels.

### 2.4 Statistical analysis

Continuous variables were expressed as mean ± standard deviation (x¯ ± s) or median (interquartile range, IQR) [M(IQR)] following normality testing, while categorical variables were reported as frequency and percentage (n, %). Intergroup comparisons were performed using the Kruskal-Wallis H test (for non-normally distributed continuous variables) or the chi-square test/Fisher’s exact test (for categorical variables). Binary logistic regression *analysis* was used to identify risk factors for severe IMLI (G3/4). Statistical significance was defined as a two-tailed *P* < 0.05. Statistical analyses were performed using SPSS software version 22.0 (IBM Corporation, Armonk, NY, USA).

## 3 Results

### 3.1 Baseline characteristics and clinical parameters of liver injury pattern groups

A total of 373 patients were included in the study (As shown in **Table 1**), with a median age of 65 years (range: 56–71 years), and 74.8% were male. The Eastern Cooperative Oncology Group Performance Status (ECOG PS) score indicated that the majority of patients (98.9%) had a score of 0-1. Among the patients, 348 (93.3%) received programmed cell death protein 1 (PD-1) inhibitors, 24 (6.4%) received programmed cell death ligand 1 (PD-L1) inhibitors, and 1 (0.3%) received a PD-1/vascular endothelial growth factor (VEGF) inhibitor. The included tumor types were primarily lung cancer (182/373, 48.8%) and digestive system cancers (147/373, 39.4%), followed by head and neck cancers (22/373, 5.9%), renal and urinary tract cancers (14/373, 3.8%), and other tumor types (2.1%). Baseline liver metastases were present in 20.1% of patients. The median values of baseline white blood cell count, absolute neutrophil count, absolute lymphocyte count, and neutrophil-to-lymphocyte ratio (NLR) were 6.3×10^9^/L, 4.1×10^9^/L, 1.4×10^9^/L, and 3.0, respectively. Median baseline values were as follows: ALT, 25 U/L; AST, 26 U/L; ALP, 90 U/L; GGT, 45 U/L; TBIL, 11.2 μmol/L; DBIL, 2.2 μmol/L.

**Table 1:** Baseline characteristics and clinical parameters of liver injury pattern groups.

Characteristic	Overall N=373	Hepatocellular n =64, 17.2%	Mixed n =159, 42.6 %	Cholestatic n =150, 40.2%	*P*
Age at diagnosis (years), Median (IQR)	65 (56-71)	61.5 (52-68)	65 (56-71)	66 (56-72)	0.026^*^
Sex, n(%)					0.213
Male	279 (74.8%)	17 (26.6%)	33 (20.8%)	44 (29.3%)	
Female	94 (25.2%)	47 (73.4%)	126 (79.2%)	106 (70.7%)	
BMI (kg/m2), Median (IQR)	22.3 (20.3-24.4)	21.9 (20.3-24.6)	22.4 (20.5-24.4)	22.2 (20.0-24.3)	0.793
Medical history, n(%)					
Thyroid diseases	22 (5.9%)	3 (4.7%)	12 (7.5%)	7 (4.7%)	0.565
Rheumatic connective tissue diseases	8 (2.1%)	0 (0%)	3 (1.9%)	5 (3.3%)	0.352
Tuberculosis	9 (2.4%)	1 (1.6%)	2 (1.3%)	6 (4.0%)	0.292
Chronic pulmonary diseases	27 (7.2%)	4 (6.3%)	11 (6.9%)	12 (8.0%)	0.897
Cardiovascular diseases	24 (6.4%)	2 (3.1%)	9 (5.7%)	13 (8.7%)	0.343
Hypertension	112 (30.0%)	14 (21.9%)	56 (35.2%)	42 (28.0%)	0.113
Diabetes mellitus	49 (13.1%)	5 (7.8%)	25 (15.7%)	19 (12.7%)	0.279
Chronic renal insufficiency	16 (4.3%)	2 (3.1%)	8 (5.0%)	6 (4.0%)	0.839
Pre-existing liver disease, n(%)					
Liver metastasis	75 (20.1%)	9 (14.1%)	26 (16.4%)	40 (26.7%)	0.032^*^
Viral hepatitis	40 (10.7%)	8 (12.5%)	15 (9.4%)	17 (11.3%)	0.761
Fatty liver	30 (8.0%)	6 (9.4%)	12 (7.5%)	12 (8.0%)	0.902
Autoimmune liver diseases	1 (0.3%)	0 (0.0%)	0 (0.0%)	1 (0.7%)	0.574
ECOG PS score, n(%)					
0	81 (21.7%)	18 (28.1%)	35 (22.0%)	28 (18.7%)	0.305
1	288 (77.2%)	46 (71.9%)	124 (78.0%)	118 (78.7%)	0.530
2	3 (0.8%)	0 (0.0%)	0 (0.0%)	3 (2.0%)	0.142
3	1 (0.3%)	0 (0.0%)	0 (0.0%)	1 (0.7%)	0.574
Baseline blood parameters, Median (IQR)					
White blood cells (×10^9^/L)	6.3 (5-7.9)	5.7 (4.5-7.7)	6.4 (5.0-7.7)	6.3 (5.0-8.4)	0.236
Neutrophils (×10^9^/L)	4.1 (3.1-5.7)	3.8 (2.8-5.3)	4.1 (3.3-5.9)	4.3 (3.1-5.9)	0.244
Lymphocytes (×10^9^/L) NLR	1.4 (1-1.7) 3 (2.1-4.9)	1.3 (1.0-1.6) 3.0 (2.1-4.7)	1.4 (1.0-1.8) 3.0 (2.1-4.7)	1.4 (1.0-1.7) 3.2 (2.3-5.0)	0.387 0.647
Baseline hepatic biochemistries, Median (IQR)					
ALT(U/L)	25 (16-38)	23.5 (15.3-36.8)	26 (17-41)	24 (16-36.3)	0.390
AST(U/L)	26 (21-35)	25.0 (21.3-33.8)	26 (21-35)	28 (21-37)	0.723
ALP(U/L)	90 (67-126)	86.5 (57.3-102.5)	81 (61-112)	110 (77.5-147)	<0.001^***^
GGT(U/L)	45 (24-83)	39.5 (23.3-57.5)	43 (23-75)	55 (27.5-94.5)	0.004^**^
TBIL (μmol/L)	11.2 (8.4-14)	11.2 (8.2-14.4)	10.8 (8.3-13.9)	11.7 (8.7-14.8)	0.423
DBIL (μmol/L)	2.2 (1.5-3.1)	2.0 (1.6-2.8)	2.1 (1.3-3.1)	2.3 (1.7-3.2)	0.120
Type of ICI, n(%)					
Anti-PD-1	348 (93.3%)	62 (96.9%)	147 (92.5%)	139 (92.7%)	0.495
Anti-PD-L1	24 (6.4%)	2 (3.1%)	11 (6.9%)	11 (7.3%)	0.547
Anti-PD-1/VEGF	1 (0.3%)	0 (0.0%)	1 (0.6%)	0 (0.0%)	1.000
Clinical Manifestations, n(%)					
Fatigue	59 (15.8%)	25 (39.1%)	18 (11.3%)	16 (10.7%)	<0.001^***^
Abdominal distension	22 (5.9%)	9 (14.1%)	7 (4.4%)	6 (4.0%)	0.010^*^
Nausea/Vomiting	17 (4.6%)	1 (1.6%)	7 (4.4%)	9 (6.0%)	0.407
Jaundice	23 (6.2%)	3 (4.7%)	10 (6.3%)	10 (6.7%)	0.920
Hepatic biochemistries during IMLI occurrence, Median (IQR)					
ALT(U/L)	109 (71.5-189)	288.5 (185.8-510.8)	120 (84-174)	75 (55.8-109)	<0.001^***^
AST(U/L)	88 (56-149.5)	182.5 (114.8-557.3)	87 (60-136)	66.5 (48-117.3)	<0.001^***^
GGT (U/L)	134 (98.5-236)	109.5 (78.3-148.5)	117 (86-156)	202 (133-459.5)	<0.001^***^
ALP(U/L)	115 (58.5-302)	102.5 (52.5-180.5)	88 (50-203)	192.5 (78-526.5)	<0.001^***^
TBIL(μmol/L)	13.8 (9.6-21.3)	14.1 (9.3-22.3)	12.8 (8.8-19.4)	15.3 (10.8-26.3)	0.014^*^
DBIL(μmol/L)	3.1 (1.9-6.6)	3.1 (1.9-7.4)	2.7 (1.7-4.9)	3.7 (2.1-9.2)	0.002^**^
Severity (CTCAE), n(%)					
1	201 (53.9%)	12 (18.8%)	99 (62.3%)	90 (60.0%)	<0.001^***^
2	94 (25.2%)	20 (31.3%)	41 (25.8%)	33 (22.0%)	0.352
3	67 (18.0%)	26 (40.6%)	17 (10.7%)	24 (16.0%)	<0.001^***^
4	10 (2.7%)	6 (9.4%)	2 (1.3%)	2 (1.3%)	0.005^**^
5	1 (0.3%)	0 (0.0%)	0 (0.0%)	1 (0.7%)	0.574
Other irAEs, n(%)					
Interstitial pneumonia	12 (3.2%)	1 (1.6%)	6 (3.8%)	5 (3.4%)	0.859
Myocarditis	6 (1.6%)	1 (1.6%)	5 (3.1%)	0 (0.0%)	0.069
Diarrhea	19 (5.1%)	1 (1.6%)	8 (5.0%)	10 (6.7%)	0.327
Skin rash	11 (3.0%)	1 (1.6%)	2 (1.3%)	8 (5.4%)	0.090
Endocrine toxicities	27 (7.2%)	5 (7.8%)	14 (8.8%)	8 (5.4%)	0.500
Nephrotoxicity	16 (4.3%)	3 (4.7%)	7 (4.4%)	5 (3.4%)	0.829
All the other irAEs*					
Time until onset (days), Median (IQR)	107 (46.5-231.5)	88.5 (35-173.5)	107 (47-209)	123.5 (51.8-259)	0.082
Cycles of ICI infusion, Median (IQR)	8 (4-15)	7 (4-16.8)	7 (4-16)	8 (4-13.3)	0.942
Treatment with corticoids, n(%)	33 (8.8%)	11 (17.2%)	12 (7.5%)	10 (6.7%)	0.034^*^
Cumulative cocorticoid dose†, n(%)					
<0.5mg/kg	3 (0.8%)	1 (1.6%)	1 (0.6%)	1 (0.7%)	0.573
0.5-1mg/kg	9 (2.4%)	2 (3.1%)	3 (1.9%)	4 (2.7%)	0.743
1-2mg/kg	15 (4.0%)	6 (9.4%)	6 (3.8%)	3 (2.0%)	0.050
>2mg/kg	6 (1.6%)	2 (3.1%)	2 (1.3%)	2 (1.3%)	0.565
Immunosuppresant, n (%)	1 (0.3%)	0 (0.0%)	0 (0.0%)	1 (0.7%)	0.574
Intravenous immunoglobulin, n (%)	15 (4.0%)	2 (3.1%)	7 (4.4%)	6 (4.0%)	1.000
ICI rechallenge, n (%)	59 (15.8)	22 (34.4%)	20 (12.6%)	17 (11.3%)	<0.001^***^
Days until resolution to normal or baseline, Median (IQR)	16 (9-33)	21 (10-37)	13 (8-24)	18 (9-35)	0.103

Data are presented as median (IQR) or n (%). Intergroup comparisons were performed using the Kruskal-Wallis H test (for non-normally distributed continuous variables) or the chi-square test/Fisher’s exact test (for categorical variables).

IQR, interquartile range; BMI, body mass index; ECOG PS, Eastern Cooperative Oncology Group performance status. NLR, absolute lymphocyte count, and neutrophil-to-lymphocyte ratio; ALT, alanine aminotransferase; AST, aspartate aminotransferase; ALP, alkaline phosphatase; GGT, gamma-glutamyl transferase; TBIL, total bilirubin; DBIL, direct bilirubin; ICI, immune checkpoint inhibitor; PD-1, programmed cell death protein 1; PD-L1, programmed cell death ligand 1; VEGF, vascular endothelial growth factor. IMLI, immune-mediated liver injury; CTCAE, common terminology criteria for adverse events; irAEs, immune-related adverse events. *P<0.05, **P<0.01, ***P<0.001.

**Figure 1:**
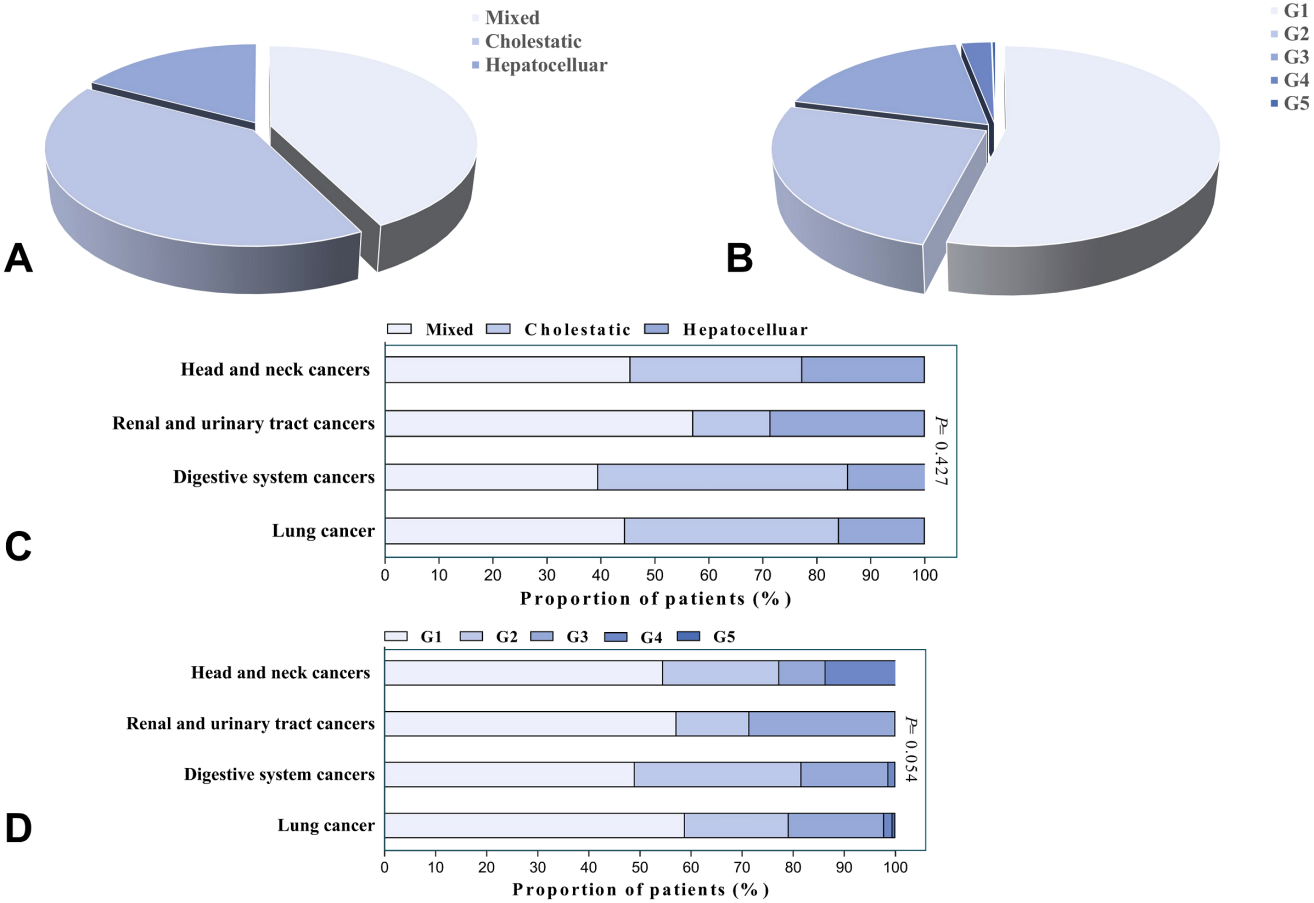
**(A)** Distribution of patients by liver injury patterns (N=373). **(B)** Distribution of patients by severity grades (N=373). Data are presented as percentages of total cases. **(C)** Distribution of liver injury patterns across cancer types (n=365). **(D)** Distribution of severity grades across cancer types (n=365). Intergroup comparisons were performed using the chi-square test/Fisher’s exact test.

The predominant liver injury patterns were mixed type (42.6%) and cholestatic type (40.2%), with hepatocellular injury being the least common (17.2%) (As shown in **Figure 1A**). No significant differences were observed among the three groups in terms of sex ratio, incidence of concurrent irAEs in other systems, time from immunotherapy initiation to liver injury onset, or cycles of ICI infusion. Patients with hepatocellular injury exhibited a significantly lower median age compared to those with cholestatic injury (61.5 vs. 66 years, *P*=0.026). Patients with elevated baseline ALP and GGT were more likely to develop cholestatic injury (*P*<0.05), and this group had a higher proportion of liver metastases compared to other types (*P*<0.05). Glucocorticoid treatment and ICI rechallenge were primarily applied in hepatocellular-type patients (*P*<0.05). The median liver function recovery time in hepatocellular injury patients (21 days) exceeded that of mixed-type (13 days) and cholestatic-type (18 days) groups; however, this difference lacked statistical significance (*P*=0.103). The severity of IMLI was associated with liver injury patterns, with hepatocellular-type patients more likely to develop severe liver injury compared to mixed-type and cholestatic-type patients (50.0% vs. 12.0% vs. 17.3%, *P*<0.001). Additionally, hepatocellular-type patients were more prone to experience clinical symptoms such as fatigue and abdominal distension (*P*<0.05) (As shown in **Table 1**).

Further analysis revealed that mixed-type injury predominated in lung cancer and head and neck cancer patients (44.5% and 45.5%, respectively), while cholestatic injury was more common in digestive system cancer patients (46.3%). Among renal and urinary tract cancer patients, mixed-type injury accounted for 57.1% of cases. Overall, no significant differences in liver injury patterns were observed across tumor types (*P*=0.427) (As shown in **Figure 1C**).

### 3.2 Clinical characteristics among patients with different grades of liver injury

In this study, the severity of IMLI was graded according to the CTCAE 5.0 criteria. The results showed that the proportions of patients with G1-G5 grades were 53.9%, 25.2%, 17.9%, 2.7%, and 0.3%, respectively (As shown in **Figure 1B**). Among them, mild liver injury (G1/2) accounted for 79.3%, while severe liver injury (G3/4) accounted for 20.7% (As shown in **Table 2**). The G5 case was excluded from further analysis due to the small sample size (n=1). There were no significant differences in demographic characteristics (age, gender), immunotherapy regimens (PD-1/PD-L1), prior treatment history, or the incidence of other systemic irAEs between patients with mild liver injury (G1/2) and severe liver injury (G3/4) (*P*>0.05). The median time from immunotherapy initiation to IMLI onset (106 days vs. 115 days, *P*=0.069) and the median number of treatment cycles (7 cycles vs. 8 cycles, *P*=0.651) also showed no statistically significant differences between the two groups. However, the liver function recovery time was significantly longer in the severe liver injury group (14 days vs. 23 days, *P*<0.05). Patients with higher baseline absolute lymphocyte counts and lower NLR had a significantly increased risk of severe liver injury (*P*<0.05). Compared to the mild group, the severe group showed lower baseline DBIL levels (*P*<0.05) and significantly higher proportions of patients receiving glucocorticoids (2.4% vs. 32.5%, *P*<0.001), intravenous immunoglobulin therapy (2.7% vs. 7.8%, *P*<0.05), and ICI rechallenge (11.2% vs. 33.8%, *P*<0.001). In the entire cohort, 93.8% (350/373) of patients showed improvement in liver function, while unresolved cases included 4 patients who voluntarily discharged themselves, 1 death, and 7 were lost to follow-up.

**Table 2:** Clinical and biological characteristics and treatment outcomes between the two study groups.

	G1/2 n=295, 79.3%	G3/4 n=77, 20.7%	*P*
Age at diagnosis (years), Median (IQR)	64 (56-70)	66 (56-71.5)	0.199
Sex, n (%)			0.873
Male	221 (74.9%)	57 (74.0%)	
Female	74 (25.1%)	20 (26.0%)	
BMI (kg/m^2^), Median (IQR)	22.41 (20.18-24.46)	21.91 (20.73-23.775)	0.574
Medical history, n (%)			
Viral hepatitis	32 (10.8%)	8 (10.4%)	0.908
Hypertension	93 (31.5%)	19 (24.7%)	0.243
Diabetes mellitus	42 (14.2%)	7 (9.1%)	0.234
Liver metastasis, n (%)	60 (20.3%)	14 (18.2%)	0.673
Baseline blood parameters, Median (IQR)			
White blood cells (×10^9^/L)	6.2 (5-7.8)	6.3 (4.7-8.05)	0.656
Neutrophils (×10^9^/L)	4.18 (3.14-5.7)	4.07 (3.02-5.220)	0.368
Lymphocytes (×10^9^/L)	1.33 (0.97-1.68)	1.47 (1.13-1.815)	0.031^*^
NLR	3.18 (2.18-5.01)	2.77 (1.88-4.015)	0.035^*^
Baseline hepatic biochemistries, Median (IQR)			
ALT (U/L)	26 (17-37)	22 (14.5-39)	0.393
AST (U/L)	27 (21-35)	25 (21-34)	0.751
ALP (U/L)	89 (67-128)	95 (72.5-122)	0.527
GGT (U/L)	46 (24-85)	44.5 (25.25-63.75)	0.464
TBIL (μmol/L)	11.3 (8.6-14.6)	10 (8.05-13.5)	0.092
DBIL (μmol/L)	2.3 (1.6-3.2)	1.9 (1.2-2.4)	0.006^**^
Type of ICI, n (%)			
Anti-PD-1	274 (92.9%)	73 (94.8%)	0.730
Anti-PD-L1	20 (6.8%)	4 (5.2%)	0.808
Previous treatment regimens, n (%)			
Chemotherapy	194 (65.8%)	56 (72.7%)	0.246
Chemotherapy plus radiotherapy	12 (4.1%)	5 (6.5%)	0.364
Chemotherapy plus targeted therapy	14 (4.7%)	5 (6.5%)	0.535
Chemotherapy plus anti-angiogenic therapy	16 (5.4%)	1 (1.3%)	0.216
None	24 (8.1%)	6 (7.8%)	0.921
Clinical Manifestations, n (%)			
Fatigue	22 (7.5%)	37 (48.1%)	<0.001^***^
Abdominal distension	6 (2.0%)	16 (20.8%)	<0.001^***^
Nausea/Vomiting	12 (4.1%)	5 (6.5%)	0.364
Jaundice	9 (3.1%)	13 (16.9%)	<0.001^***^
Hepatic biochemistries during IMLI occurrence, Median (IQR)			
ALT (U/L)	94 (67-140)	331 (190.5-533)	<0.001^***^
AST (U/L)	72 (54-117)	271 (144-569)	<0.001^***^
GGT (U/L)	123 (90-182)	275 (151-578)	<0.001^***^
ALP (U/L)	89 (50-202)	361 (165.5-646.5)	<0.001^***^
TBIL (μmol/L)	12.7 (8.8-18.3)	20.1 (14.65-44.2)	<0.001^***^
DBIL (μmol/L)	2.7 (1.7-4.80)	7.5 (3.3-28.35)	<0.001^***^
Pattern of liver injury, n (%)			
Hepatocellular	32 (50.0%)	32 (50.0%)	1.000
Mixed	140 (88.1%)	19 (11.9%)	<0.001^***^
Cholestatic	123 (82.6%)	26 (17.4%)	<0.001^***^
Other irAEs, n (%)			
Interstitial pneumonia	9 (3.1%)	3 (3.9%)	0.991
Myocarditis	5 (1.7%)	1 (1.3%)	1.000
Diarrhea	15 (5.1%)	4 (5.2%)	1.000
Skin rash	7 (2.4%)	4 (5.2%)	0.355
Endocrine toxicities	20 (6.8%)	7 (9.1%)	0.486
Nephrotoxicity	13 (4.4%)	2 (2.6%)	0.694
Time until onset (days), Median (IQR)	106 (41-220)	115 (70-287)	0.069
Cycles of ICI infusion, Median (IQR)	7 (4-15)	8 (3-16.5)	0.651
Treatment with corticoids, n (%)	7 (2.4%)	25 (32.5%)	<0.001^***^
Cumulative corticoid dose†, n (%)			
<0.5mg/kg	2 (0.7%)	1 (1.3%)	0.502
0.5-1mg/kg	0 (0%)	9 (11.7%)	<0.001^***^
1-2mg/kg	5 (1.7%)	10 (13.0%)	<0.001^***^
>2mg/kg	0 (0%)	5 (6.5%)	<0.001^***^
Immunosuppressant, n (%)	0 (0%)	1 (1.3%)	0.207
Intravenous immunoglobulin, n (%)	8 (2.7%)	6 (7.8%)	0.037^*^
ICI rechallenge, n (%)	33 (11.2%)	26 (33.8%)	<0.001^***^
Days until resolution to normal or baseline, Median (IQR)	14 (8-29)	23 (12-39)	0.007^**^

†All glucocorticoid doses are expressed as methylprednisolone equivalents. Data are presented as median (IQR) or n (%). Intergroup comparisons were performed using the Kruskal-Wallis H test (for non-normally distributed continuous variables) or the chi-square test/Fisher’s exact test (for categorical variables).

IQR, interquartile range; BMI, body mass index; NLR, absolute lymphocyte count, and neutrophil-to-lymphocyte ratio; ALT, alanine aminotransferase; AST, aspartate aminotransferase; ALP, alkaline phosphatase; GGT, gamma-glutamyl transferase; TBIL, total bilirubin; DBIL, direct bilirubin; ICI, immune checkpoint inhibitor; PD-1, programmed cell death protein 1; PD-L1, programmed cell death ligand 1. IMLI, immune-mediated liver injury; irAEs, immune-related adverse events. ^*^ *P*<0.05, ^**^ *P*<0.01, ^***^ *P*<0.001.

Further analysis revealed no significant differences in the severity of IMLI across cancer types (*P*=0.054) (As shown in **Figure 1D**).

### 3.3 Correlation analysis between peripheral blood immune cell subsets and IMLI severity

This study monitored peripheral blood immune cell subset counts at baseline, early ICI treatment phase, and recovery phase. Patients with severe IMLI (G3/4) exhibited significantly higher baseline NK cell counts (median [IQR]: 347.2 [210-524.1] vs. 219.7 [107.6-362.6] cells/μL, *P*=0.030) and elevated recovery-phase total T cells (CD3^+^ median [IQR]: 1121.28 [793.271-1454.594] vs. 781.87 [523.44-1140.36] cells/μL, *P*=0.043) and CD8^+^ T cells (median [IQR]: 524.9 [272.56-835.751]vs. 270.68 [195.63-448.79] cells/μL, *P*=0.026) compared to mild cases (G1/2) (As shown in **Table 3**).

**Table 3:** Peripheral blood immunological parameters between the two study groups.

	G1/2 n=67, 84.8%	G3/4 n=12, 15.2%	*P*
Baseline immune cell counts, Median (IQR)			
Total CD3^+^ T cells (/μL)	897.1 (522.3-1185.6)	971.4 (792.7-1134.7)	0.521
CD4^+^ T cells (/μL)	547.7 (250.2-667.9)	530.9 (440.9-706.3)	0.662
CD8^+^ T cells (/μL)	313 (191.3-483.8)	341.5 (257.9-510.6)	0.495
CD4/CD8	1.5 (0.9-2.4)	1.4 (0.8-2.1)	0.764
NK cells (/μL)	219.7 (107.6-362.6)	347.2 (210-524.1)	0.030^*^
B cells (/μL)	121.5 (74.9-186.6)	120.8 (95.1-160.3)	0.557
Early phase immune cell counts, Median (IQR)	G1/2 n=24, 77.4%	G3/4 n=7, 22.6%	*P*
Total CD3^+^ T cells (/μL)	717.545 (518.238-1198.799)	944.36 (791.252-1265.75)	0.216
CD4^+^ T cells (/μL)	449.68 (278.028-642.2515)	621.57 (382.228-728)	0.253
CD8^+^ T cells (/μL)	278.293 (209.28-430.6005)	348.04 (281.93-427.5)	0.317
CD4/CD8	1.52 (1.035-2.2625)	1.32 (1.08-2.2)	0.878
NK cells (/μL)	223.86 (136.749-445.618)	449.96 (101.25-819.49)	0.473
B cells (/μL)	84.749 (48.0060-142.7135)	149.25 (59.5-241.7)	0.234
Recovery phase immune cell counts, Median (IQR)	G1/2 n=35, 72.9%	G3/4 n=13, 27.1%	*P*
Total CD3^+^ T cells (/μL)	781.87 (523.44-1140.36)	1121.28 (793.271-1454.594)	0.043^*^
CD4^+^ T cells (/μL)	378.19 (220.85-714.72)	431.683 (378.66-602.330	0.521
CD8^+^ T cells (/μL)	270.68 (195.63-448.79)	524.9 (272.56-835.751)	0.026^*^
CD4/CD8	1.3 (0.83-1.96)	1.06 (0.435-1.885)	0.164
NK cells (/μL)	149.76 (83.85-297.5)	212.553 (101.336-654.591)	0.133
B cells (/μL)	92.752 (45.13-145.92)	95.238 (71.9-157.72)	0.521

Intergroup comparisons were performed using the Kruskal-Wallis H test. IQR, interquartile range; NK, natural killer. ^*^ *P*<0.05.

### 3.4 Risk factors for IMLI

**Figure 2:**
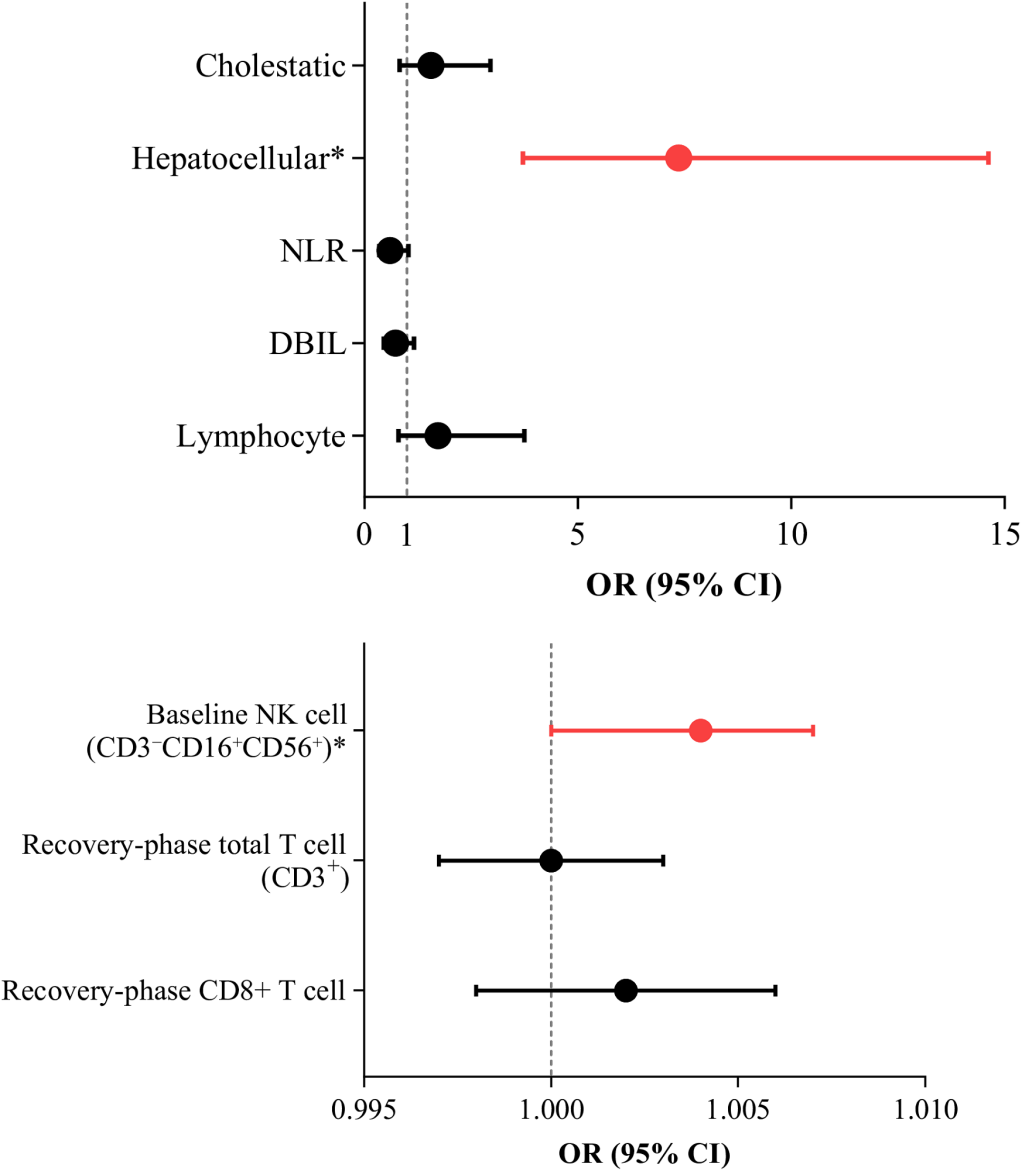
Risk Factors for Severe Liver Injury. Binary logistic regression analysis was used to identify risk factors for severe IMLI (G 3/4).

The hepatocellular injury was identified as an independent predictor of severe IMLI (*OR*=7.368, 95% CI: 3.713-14.622; *P*<0.001), while baseline high NK cell counts showed a weaker predictive effect (*OR*=1.004, 95% CI: 1.000-1.007, *P*=0.036). Although univariate analysis revealed associations between recovery-phase total T cell counts (CD3^+^), CD8^+^ T cell counts, and the severity of liver injury, these associations were not significant in multivariate regression analysis (*OR*=1.000, *P*=0.916; *OR*=1.002, *P*=0.350) (As shown in **Figure 2**).

### 3.5 Temporal analysis of IMLI

**Figure 3:**
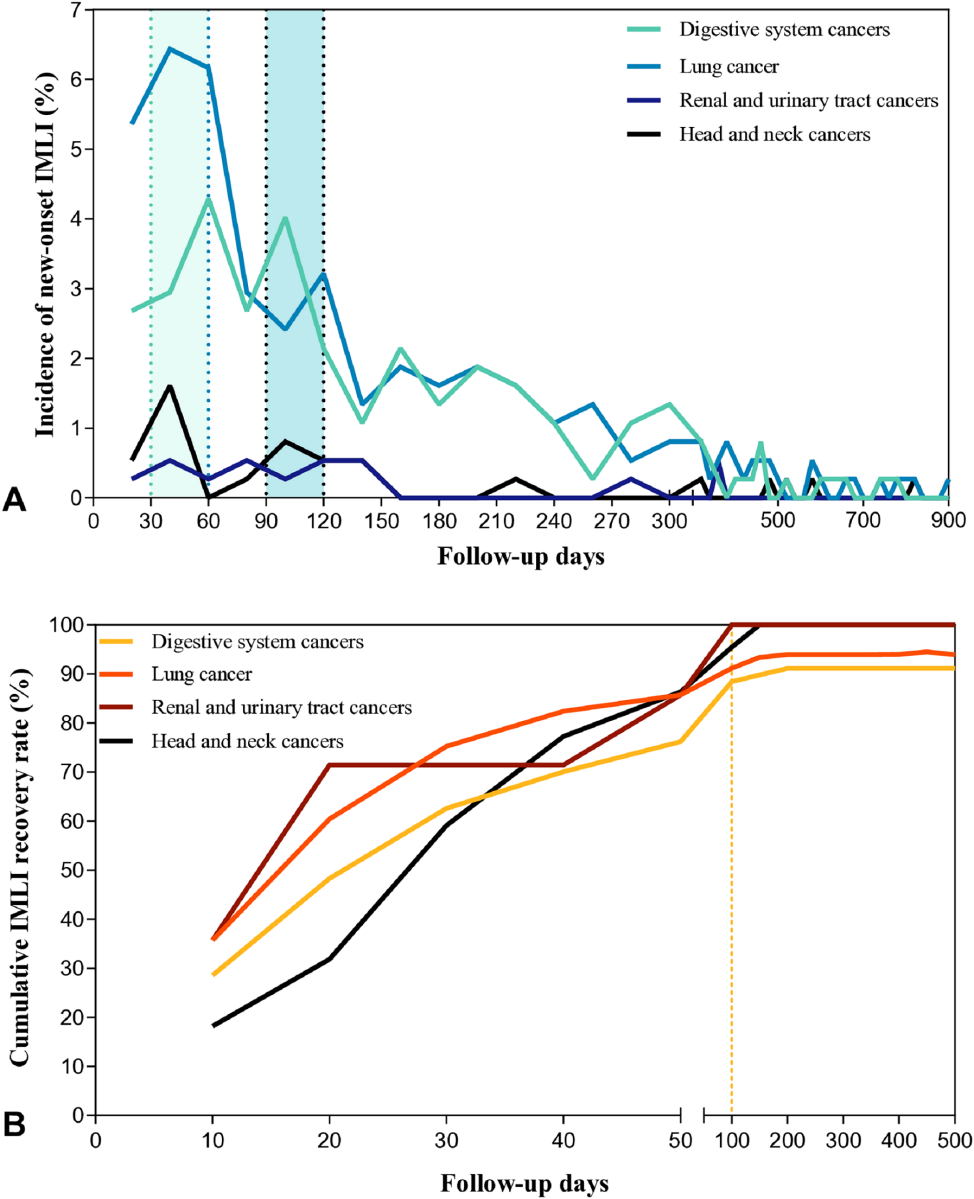
**(A)** Incidence of New-onset IMLI Across Cancer Types. **(B)** Cumulative Recovery Rate of IMLI Across Cancer Types. Patients were followed until July, 2024. Patients lost to follow-up or who died were censored at the last contact date. Incidence of new-onset IMLI = Number of new IMLI cases within a specific time interval/Total study population (N=373). Cumulative recovery rate = Cumulative number of recovered IMLI cases within a specific time interval/Total study population (N=373). IMLI, immune-mediated liver injury.

This study found that the risk of IMLI in patients across various cancer types during the follow-up period exhibited a bimodal distribution. The first peak occurred within 1–2 months after the initiation of immunotherapy, followed by a secondary peak at 3–4 months. The risk of new-onset IMLI was extremely low after 12 months (As shown in **Figure 3A**). The cumulative recovery trends of IMLI were generally similar across cancer types, with most cases recovering within 50 days. Delayed recovery (>100 days) was rare (As shown in **Figure 3B**).

## 4 Discussion

By analyzing 373 IMLI patients treated with PD-1/PD-L1 inhibitors across different tumor types, we revealed a bimodal onset, uniform injury patterns and severity across tumor types, and NK cells as a potential predictive marker, which helps to optimize advancing personalized monitoring and universal therapeutic decision-making.

This study reveals that IMLI exhibits a bimodal temporal distribution (1–2 months vs. 3–4 months), which differs significantly from the previously reported (12) unimodal distribution (e.g., 1.4 months vs. 4.7 months). However, the overall onset window (1–4 months) remains consistent with the high-risk period for IMLI (within the first 6 months of treatment) reported in most studies (21). The observed bimodal dynamic of IMLI progression closely parallels the established mechanism of epitope spreading (ES) (22). During the initial phase, clonal expansion of tumor antigen-specific T cells (corresponding to the first IMLI peak) (23, 24) progressively evolves through ES into systemic autoreactivity against hepatic abundant self-antigens, including cytochrome P450 and mitochondrial antigens, ultimately manifesting as the second peak of liver injury (22). The elevated recovery-phase CD8^+^ T cells in severe cases (524.9 vs. 270.68 cells/μL, *P*=0.026) further support this mechanism, corroborating single-cell evidence of accelerated effector T cell differentiation post-PD-1 blockade (25). Early diversification of the T-cell repertoire after ICI initiation indirectly supports this autoimmune cascade process (26). This temporal stratification advocates for risk-adapted monitoring within the first 4 months, aligning surveillance intensity with peak hazard periods. Despite the significant acute-phase injury, the disease exhibits self-limiting characteristics due to CD8^+^ T cell exhaustion **(Tex)** in the chronic antigen environment (27) and liver parenchymal regeneration mediated by hepatic progenitor cells (28). Tex constitutes a complex dynamic process spanning from precursor exhausted T cells with stem-like proliferative capacity to terminally exhausted T cells that completely lose effector functions and proliferative potential. This process not only represents a crucial immunoregulatory mechanism but also serves as a major pathway for tumor resistance to immune checkpoint blockade therapy. As established immune checkpoint receptors, PD-1 and T-cell immunoglobulin and mucin-domain containing-3 (TIM-3) represent reliable indicators of exhausted T cells (29). Their persistent overexpression on T cells results in the suppression of effector functions, diminished proliferative capacity, and attenuated cytokine production, which are consistently observed in both tumor microenvironments and chronic inflammatory states (30, 31). A comprehensive understanding of the multidimensional features of Tex holds significant implications for developing novel immunotherapeutic strategies.

Our study observed pan-cancer uniformity in liver injury patterns (*P*=0.427) and severity (*P*=0.054), suggesting a systemic immune dysregulation rather than tumor-driven pathology. Mechanistically, the PD-1/PD-L1 signaling pathway is pivotal to hepatic immune homeostasis (32, 33). Under physiological conditions, constitutive PD-L1 expression on liver sinusoidal endothelial cells and hepatocytes suppresses CD8^+^ T cell activation via TCR-peptide-major histocompatibility complex (TCR-p-MHC) signaling blockade, maintaining peripheral tolerance through T cell exhaustion and apoptosis (34). ICI-mediated disruption of this pathway unleashes autoreactive CD8^+^ T cells, which infiltrate the liver parenchyma and drive injury via cytotoxic effector gene activation, including perforin and granzyme (23, 24) while secreting pro-inflammatory cytokines like IFN-γ and TNF-α to recruit Kupffer cells and amplify inflammation through a self-reinforcing loop (35–37). The liver’s rich self-antigen repertoire, including cytochrome P450 and mitochondrial antigens, likely facilitates cross-cancer T cell targeting, explaining the observed clinical homogeneity (38). These findings align with the review demonstrating tumor-agnostic irAE profiles for ICIs (39), contrasting with emerging tumor-specific irAE prediction models (18) and meta-analysis (17). The homogeneous sample composition (predominantly lung and digestive system cancers) and treatment protocols likely contributed to the absence of observed differences. Future multicenter prospective studies with diverse cancer types are warranted for validation.

Predictive biomarkers are crucial for guiding treatment decisions (40). To our knowledge, this is the first study to identify baseline NK cell counts as a potential biomarker for severe IMLI (*OR*=1.004, *P*=0.036). Although statistically significant, the interpretation of this finding requires caution, given that the *OR* is close to 1, and the clinical significance of NK cells remains incompletely understood. Multiple studies have demonstrated the critical role of NK cells in antitumor immunity (3, 41–43). As key components of the innate immune system, NK cells can induce target cell apoptosis through the release of perforin, granzymes, and expression of Fas ligand (FasL) and TNF-related apoptosis-inducing ligand (TRAIL). Additionally, they can secrete chemokines such as TNF-α and CCL3/4/5, recruiting more immune cells to infiltrate the liver and exacerbate tissue damage. Thus, a high baseline NK cell population may be more prone to excessive activation, leading to severe IMLI. On the other hand, NK cells can be classified into CD56bright and CD56dim subsets based on specific surface markers. The CD56dim NK cell subset primarily exerts cytotoxic effects by directly killing hepatocytes, whereas the CD56bright NK cell subset not only secretes pro-inflammatory cytokines that aggravate liver injury but also transmits inhibitory signals through high expression of natural killer group 2A (NKG2A), potentially suppressing excessive inflammation via IL-10 secretion (44, 45). Thus, NK cells may play dual roles in IMLI progression by initially exacerbating tissue damage and subsequently suppressing excessive immune responses through regulatory functions. However, baseline total NK cell counts fail to distinguish functional subset differences, thereby attenuating the effect size. Future studies should further delineate the proportions of NK cell functional subsets and their specific roles in IMLI through larger prospective cohorts to validate these findings.

Despite the significant findings, this study has certain limitations. First, as a single-center retrospective study, it is inherently subject to selection and information biases, and some subgroups had limited sample sizes. Second, peripheral blood immune cell profiling fails to capture hepatic microenvironmental dynamics, compounded by the lack of large-scale histopathological data. The absence of longitudinal multi-timepoint immune monitoring further restricts the analysis of temporal immune cell evolution in IMLI pathogenesis. Third, the predominance of lung and digestive system cancers may mask tumor-specific differences. Future studies should integrate multi-omics approaches to clarify local immune dynamics further, providing references for developing targeted intervention strategies with specific regulatory mechanisms.

## 5 Conclusions

Our findings advocate for risk-adapted monitoring with tailored surveillance windows focusing on the first 4 months post-IC, with intensified surveillance during bimodal peaks. The pan-cancer consistency of IMLI characteristics supports standardized management protocols across malignancies. Furthermore, baseline NK cell counts may serve as a potential biomarker to refine risk-stratification strategies. Future studies are warranted to establish clinically actionable cut-off values for optimized patient management.

## Data Availability

The raw data supporting the conclusions of this article will be made available by the authors, without undue reservation.
